# The Book World of Medicine and Science

**Published:** 1897-08-28

**Authors:** 


### The Book World of Medicine and Science.

Homburg and its Waters, with articles on Obesity, Gout, Weak Heart, &c., &c., by N. E. Yorke-Davies. (London: Sampson Low, Marston, and Co. 1897. Price 1s. 6d.)

This is a little book containing only a very little about Homburg. Perhaps there is not very much to be said on the subject; at any rate, only a small part even of the one chapter of twenty-one pages nominally devoted to it is really given up to either Homburg or its waters. The rest of the book consists of a little catchy talk about obesity, gout, weak heart, &c., all dealt with in the author's own peculiar manner, and a few very vague suggestions about diet. The book is clearly not written for the medical profession, and to patients we cannot recommend it.

Notes on Practical Sanitary Science. By William H. Maxwell, Assistant Engineer and Surveyor, Leyton Urban District Council. Part I.—Ventilation, Heating, and Lighting. Forty-six Illustrations and Frontispiece. Eighty-nine pages. (London: Sanitary Publishing Company, Limited, 5, Fetter Lane, Fleet Street, E.C. 1897.)

This book, reprinted from papers published in the *Sanitary Record*, is the first of a series written from a sanitary engineer's point of view, and dealing, in addition to ventilation, light, and heat, with the questions of calculation of areas, cubic space, &c., removal and disposal of town refuse, water and water supply, plumber's work, &c. The series is intended to be of service to candidates preparing for professional certificates such as those granted by the Association of Municipal and County Engineers and the Sanitary Institute. The book does not pretend to be an exhaustive treatise. It is, however, an entirely useful book, being both lucid and condensed, and the diagrams and illustrations are excellent. It is fully up to date, and well supplied with references, and as a text book is admirably suited to the needs of those for whom it is intended.

Oban: A Health and Holiday Resort. By Edwin Baily, M.B., C.M., Medical Officer of Health for the Burgh.

This is a most attractive little souvenir, and is likely to tempt many to visit the popular watering-place of which it treats. The growth of Oban has been considerable, but still it remains a comparatively small place, its population being somewhat over 5,000. To the visitor this is a great mercy, inland attractions being accessible within reasonable distance as well as those provided by the sea front. Dr. Baily enters into many details as to the climatology of the place, which evidently obtains the full advantage to be derived from its position on the West Coast and its exposure to the influence of the Gulf Stream. It has, however, a high rainfall, amounting to 52 inches in the year, there being 230 days on which a registerable amount of rain fell. We are assured, however, that although days of heavy driving rain are sometimes experienced, a sudden sharp downpour is the rule, the sun quickly bursting out again when the shower has passed. The percentage of humidity seems to be lower than at some other places. It is well to know that, although so generally visited in the summer holiday season, Oban is most beautiful in spring months and in the late autumn. Much emphasis is also laid upon its advantages as a winter health resort.

Franzenbad: An Austrian Health Resort. Published by the Health Committee, 1897. (London: Baillière, Tindall, and Cox.)

It is necessary nowadays that every watering-place should keep its attractions well before the public eye. Hence, many little books. At this we do not grumble; nay, we rejoice, for it cannot be denied that so gregarious is mankind and so exacting is fashion that we tend to run in ruts, and that the ruts become desperately crowded. Hence the health-seeking public, and even those whose search only extends to recreation, may well be glad to have the advantages of the less known watering-places brought before them. Not that the alkaline, saline, and ferruginous waters of Franzenbad are not well-known enough in their own country. But they certainly deserve to be better known than they are to English wanderers. This little book of 90 pages, full, as it is, of pictures, maps, and information, is, then, well worth referring to. It is stated that the appliances at Franzenbad are useful in "catarrhs" of all the mucous canals, in gout, in fatness, in scrofula and anæmia, and many other conditions.

Diet Chart and Alimentary Index. Suggested by M. J. Kenny, L.R.C.P.Ed. (Bristol: John Wright and Co. 1897. Price 1s. 6d.)

This is an attempt to facilitate exactitude in prescribing diet. We cannot but feel, however, that the exactitude is dearly purchased at the expense of the curtailment of choice of foods, which the diet charts would seem to indicate. The alimentary index consists of a list of a few diseases, to each of which the appropriate articles of diet are appended. We may be allowed to have our doubts as to the utility of this arrangement. It is difficult to understand what can be meant by apoplexy in view of the diet prescribed, viz., "White meats, milk, ripe fruits, green vegetables," nor do we think much assistance is to be obtained from the directions given as to the diet of pernicious anæmia, which runs as follows: "Exclusively farinaceous diet.—*Hunter*. Milk, eggs, scraped raw beef, meat juice.—*Mott*." As for the diet charts, which are to be torn out and given to the patients, the list of foods (to be ticked off as in a washing bill) is distinctly meagre, and oddly enough does not contain all the articles mentioned in the alimentary index.

Left-Hand Writing. John Jackson, F.E.I.S. (London: Sampson, Low, Marston, and Co., 1897.)

Messrs. Sampson Low, Marston, and Co have just published, and Mr. John Jackson, F.E.I.S., written, an interesting book on "Left-Hand Writing." The volume has been prepared for the acquirement of a free and hygienic style of left-hand penmanship, and was "produced at the suggestion of Dr. W. R. Gowers." Many years before this the subject of left-handed writing and ambidexterity in the art had been ventilated, the author tells us, by himself, "both by correspondence and interviews. During the preparation of this unique manual repeated references to medical authorities, including the above-named gentleman, and consultations with many left-handed and ambidextrous persons have been made and secured, in order that it might, as far as possible, be constructed in strict accordance with physiological as well as with calligraphic requirements." The author has spared no pains in his task. The exercises as here given are arranged with reference to size in a regular order, beginning with a large size and gradually diminishing to a rather small hand, the final examples being given in a bolder and freer style. The little volume brings its suggestions. Is it not possible that the practice of writing with the left hand might develop in right-handed people the undeveloped centre on the right side of the brain corresponding to the centre for speech on the left? We know that in left-handed people this centre is on the right side of the brain. Why, therefore should not the education of the left hand, in right-handed people, develop that undeveloped centre, which controls not only our speech, but also all our powers of communicating with our fellow human beings.

We believe that, so far, no statistics have been published with regard to ambidextrous people, therefore there is certainly a probability that the practice of writing with the left hand may not only give the individual the advantage of his centres, fully developed, in case of an injury implicating one of them, for instance, but also would give us a better chance of advancing our knowledge of physiology in the one direction in which our means of advance is at present limited, namely, the localisation of the intellectual centres.

